# Comparing the impact of active floor-rise training with video demonstration on fear of falling and independent floor-rise ability in older adults living in the community: a pilot cluster randomised controlled trial

**DOI:** 10.1093/ageing/afag064

**Published:** 2026-03-27

**Authors:** Shane C Seeley, Dawn A Skelton, Chee-Wee Tan, Ben Stansfield, Philippa M Dall

**Affiliations:** School of Health & Life Sciences, Research Centre for Health (ReaCH), Glasgow Caledonian University, Glasgow, UK; School of Health & Life Sciences, Research Centre for Health (ReaCH), Glasgow Caledonian University, Glasgow, UK; Singapore Institute of Technology, Singapore, Singapore; School of Health & Life Sciences, Research Centre for Health (ReaCH), Glasgow Caledonian University, Glasgow, UK; School of Health & Life Sciences, Research Centre for Health (ReaCH), Glasgow Caledonian University, Glasgow, UK

**Keywords:** older adults, floor-rise training, exercise therapy, fear of falling

## Abstract

**Background:**

Many older adults cannot rise from the floor independently after a fall, increasing risks of long-lies and reliance on emergency services.

**Objective:**

Investigate whether floor-rise training (FRT) reduces fear of falling (FoF) and improves floor-rise ability in older adults.

**Design:**

Multi-centre, cluster-randomised controlled before-after pilot trial.

**Setting:**

Five community-based Otago exercise classes run by a third-sector organisation.

**Participants:**

Sixty-one community-dwelling older adults (aged ≥65 years) attending weekly Otago classes were randomised (FRT *n* = 27, control *n* = 34). Forty-nine completed to follow-up and were analysed (FRT *n* = 22, control *n* = 27). No adverse events occurred.

**Methods:**

Classes were cluster-randomised (3:2 allocation). Intervention: 5 weekly 20-minute FRT sessions utilising backward-chaining. Controls viewed a FRT demonstration video followed by discussion (20-minutes total), without physical practise. Primary outcome: Falls Efficacy Scale-International (FES-I). Secondary outcomes: timed floor-rise and independent floor-rise ability (from supine, side-sitting, half-kneeling), Perceived Ability to Manage Risk of a Falls or Actual Falls (PAMF), FoF and activity avoidance, measured via visual analogue scales (1-item-question).

**Results:**

Primary outcome (FES-I), FoF and activity avoidance showed no significant differences. However, FRT participants significantly reduced floor-rise times compared to controls: supine (13.1 s to 7.1 s, *P* = .001), side-sitting (8.0 s to 4.6 s, *P* = .046), and half-kneeling (3.9 s to 1.5 s, *P* < .001). Post-intervention, 100% of FRT participants could rise from supine versus 63% of controls (*P* = .007). PAMF scores increased significantly in the FRT group (13.6 to 16.3, *P* = .033).

**Conclusion:**

Although FoF did not change, a brief FRT intervention significantly improved floor-rise ability and PAMF. Integrating FRT into fall prevention programmes may reduce long-lie consequences.

## Key points

- Floor-rise training (FRT) did not reduce fear of falling or avoidance of activity in older people already attending falls prevention exercise.
- A brief FRT intervention (3–5 sessions) significantly improved older adults’ floor-rise ability and perceived post-fall recovery efficacy.
- Post-intervention, 100% of intervention participants could independently rise from supine, compared to no change in controls.
- Integrating FRT into existing fall prevention programmes may reduce long-lie associated harm and emergency service use.

Falls are a major public health concern, particularly with an ageing population [1]; 33% of adults over 65 and 50% over 80 fall annually [2], resulting in 17 million years of life lost [3]. Long-lie events (remaining on floor >1 hour after falling) are linked to delayed recovery and increased hospitalisation, admission to residential care and mortality [4–6]. Beyond the physical consequences, long-lies lead to loss of dignity, increased fear and activity avoidance, reinforcing the cycle of functional decline and increased fall risk [7, 8].

Fall-related ambulance callouts place a significant burden on emergency services, costing the UK NHS £75.5 million in 2012, projected to rise to £118.9 million by 2030 [9]. Although up to 65% of ambulance call-outs for falls are ‘lift-assists’ that require no further medical attention [10–12], the recurrence rate remains high, with 47% of these individuals re-contacting emergency services within 2 months [13].

While exercise interventions targeting strength, balance and gait reduce fall risk [14], falls will still occur, and 50% of older adults who fall cannot rise independently [9, 15]. Floor-rise ability is an important functional skill, recognised in international guidelines [16], which is rarely taught to fallers by physiotherapists or ambulance crews [8] and is frequently omitted from fall prevention programmes [16]. The Falls Management Exercise (FaME) programme is the only evidence-based exercise intervention for high-risk fallers that includes floor-rise training (FRT) [7, 17–19] but usually only after 12 weeks of the 24-week programme. FaME classes significantly reduced falls by 31% and improved floor-rise time by 10% [17, 20]. Previous research investigating FRT as a standalone intervention is limited to a few small RCTs of variable quality [21–23]. While these studies showed improvements in floor-rise ability, they often utilised high training doses (up to 36 sessions) [22, 23] or, in lower-dose studies, relied on resource-intensive one-to-one physiotherapy supervision [21]. It remains unclear if a brief, scalable, group-based intervention can achieve similar functional gains.

The relationship between FRT and fear of falling (FoF) requires investigation. FoF influences activity levels and quality of life [24], often leading to avoidance of activity and functional decline [24, 25]. Exercise-based interventions have been shown to reduce FoF [26, 27]. However, there is limited research investigating FRT’s effect on FoF, leading a systematic review to call for future investigation [28]. Although one study found a non-significant reduction [29], the theoretical rationale remains. FoF may be driven by the fear of fall consequences, such as long-lies and the physical damage from the fall. As FaME (which includes FRT) significantly reduces FoF in older adults [30], practising FRT may be a significant component of this measured reduction. Therefore, this study investigates whether practising FRT in as few as three to five sessions reduces FoF and improves floor-rise ability in older adults attending Otago exercise classes, compared to a control group watching instructional videos.

## Methods

### Study design

A multi-centre, cluster-randomised, controlled pilot trial with a parallel-group design, comparing FRT with an educational video. The allocation ratio was 3:2 (intervention: control) with unequal cluster sizes. One protocol deviation occurred: a reduction in intervention duration from 6 to 5 weeks.

### Study setting and participants

Participants (≥ 65 years old) who took part in weekly Otago exercise classes were recruited (January–February 2024) through a third sector community organisation. Otago is a falls prevention class without FRT [31]. Exclusion criteria were: requiring a walking frame to mobilise indoors, contraindications to FRT (e.g. awaiting knee replacement surgery), uncontrolled medical condition (a chronic condition, such as unstable cardiovascular disease or poorly controlled diabetes, that could compromise safety in the physical intervention), body mass index (BMI) ≥30 kg/m^2^ and an inability to comprehend instructions. Baseline characteristics were age, sex, number of falls in previous year, use of walking aid indoors/outdoors and months/years attending Otago classes. All participants provided written informed consent. Ethics approval was granted by the institutional committee, and the research was carried out in accordance with the declaration of Helsinki.

### Blinding and randomisation

An independent researcher generated a concealed randomisation sequence assigning clusters to intervention (*n* = 3) or control (*n* = 2). Participant blinding was not feasible beyond baseline testing given the nature of the intervention (i.e. either physical practice or a video demonstration). Intervention delivery, baseline and follow-up assessments were conducted by the primary researcher who was unblinded during follow-up assessments due to resource constraints, though standardised procedures were strictly followed to minimise bias.

### Intervention

The intervention comprised 5 weekly sessions (20 minutes), following Otago classes, delivered in groups of eight or fewer. Sessions utilised the backward-chaining method (BCM) [32], where participants master the movement in reverse order (see Supplementary Appendix 1 for full protocol). Participants progressed from guided technique learning to independent practice, with modifications permitted for mobility restrictions [33]. The intervention was delivered by a physiotherapy student, who is also a qualified personal trainer, with a Mangar lifting cushion (ELK, Winncare) [34] available for safety. Controls viewed the NHS Inform video ‘Upwards and Onwards’ [35], divided into segments over five 20-minute sessions including facilitated discussion. After the study was complete, control participants were offered the opportunity to complete FRT training.

### Outcome measures

Outcomes were assessed at baseline and 1-week post-intervention. No changes were made to the pre-specified primary or secondary outcomes after trial commencement.

The primary outcome was FoF, measured using the Falls Efficacy Scale-International (FES-I). The FES-I scale (16–64, higher scores indicate greater fear) has excellent test–retest reliability and validity in older adults [36]. The FES-I measures pre-fall efficacy (confidence in performing daily activities without falling), which is distinct from the post-fall management efficacy captured by a secondary outcome. Two single-item visual analogue scales (VAS) were also used to measure FoF indoors and outdoors. These were chosen as they may have a higher acceptability than FES-I due to their brevity.

Secondary outcomes included the Perceived Ability to Manage Risk of Falls or Actual Falls (PAMF), activity avoidance due to fall concerns, and floor-rise performance (time and ability).

The PAMF scale (consisting of five items rated 1–4, total score 20, higher scores indicating better post-fall efficacy) utilised in this study was developed by Tennstedt *et al.* [37]. This scale has demonstrated good internal consistency (Cronbach’s *α* = 0.76–0.84) and initial construct validity in community-dwelling older adults [37]. Its use is further justified by evidence that post-fall efficacy is a distinct domain of the fall’s efficacy construct [38, 39].

FoF has been linked with avoidance of activity and a progressive loss of health and quality of life [24]. Activity avoidance was captured using a one-item VAS scale asking: ‘How often do you avoid activities through concern about falling?’, rated from 100% of the time to 0% of the time to minimise the burden on participants.

**Figure 1:**
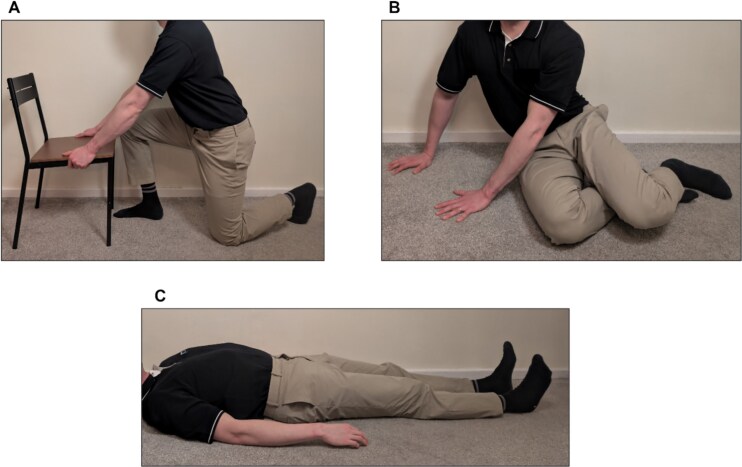
The three standardised starting positions used for the floor-rise assessment. (A) Half-kneeling; (B) Side-sitting; (C) Supine.

Floor-rise performance was evaluated using a timed test and recording independent ability. Participants were instructed to rise from half-kneeling (one knee down), side-sitting (weight on one hip) and supine (lying flat on the back) positions using a sturdy chair for support (Figure 1). Time was measured from movement initiation to standing. The binary ability to rise from each position was also recorded. While this binary ability has high clinical relevance for preventing serious fall injuries [40], it fails to capture improvements in those able to rise at baseline (a ceiling effect). In contrast, timed floor-rise performance is considered a valid measure of physical capacity [41], and floor transfer speed is a known predictor of future falls [42]. However, there is no established minimal clinically important difference for the timed floor-rise test in the literature.

While floor-rise ability without support (i.e. without using a chair) is relevant for outdoor falls [40, 43], this study focused on the assisted floor-rise. This decision was based on: (i) Ecological validity regarding long-lies, which predominantly occur indoors where furniture is available; (ii) Alignment with the FRT intervention, which specifically trained the BCM utilising a chair and (iii) Health and safety concerns, regarding fall risk during unassisted attempts.

Two focus groups (Intervention/Control) explored the research question: ‘How did participants perceive the acceptability, impact and feasibility of the FRT intervention and trial processes?’ Discussions followed a semi-structured format (see Supplementary Appendix 2 for all of the interview prompts) elucidating perspectives in a naturalistic way via group interaction [44]. The discussions covered: (i) Intervention acceptability (videos/steps of FRT); (ii) Perceived impact and (iii) Trial feasibility (recruitment/outcome measures). One researcher moderated while another recorded detailed flipchart notes. These notes, validated by participants, formed the primary data record, supplemented by key quotes extracted from audio recordings. Reflexivity was considered, acknowledging the researcher’s role in intervention delivery.

### Statistical analysis

The target sample was 36 [45]; analysis was intention-to-treat. Baseline characteristics were compared using independent t-tests or Mann–Whitney U tests. Continuous outcomes were analysed using a mixed-factorial analysis of variance (ANOVA; Group × Time), utilising pairwise comparisons and log-transformations where assumptions were violated. Significant differences (*P* < .05) were characterised using mean differences with 95% confidence intervals (CI) and effect sizes, with partial eta squared (*ƞ_p_*^2^) interpreted as: small 0.01–0.06; medium 0.06–0.14 and large >0.14 [46, 47].

Binary outcomes (ability to rise) were analysed using linear probability models (LPM) to estimate adjusted risk differences (RD). LPMs were selected to handle ‘perfect prediction’ (100% success) in the intervention group. Models were adjusted for baseline ability, age and Otago duration, utilising bootstrapping (1000 replications) for robust inference. A secondary sensitivity analysis using analysis of covariance (ANCOVA) was performed on key outcomes to account for baseline imbalances (age/Otago duration). Analysis used SPSS (version 29.0.1.0).

## Results

### Participants flow and adherence

**Figure 2:**
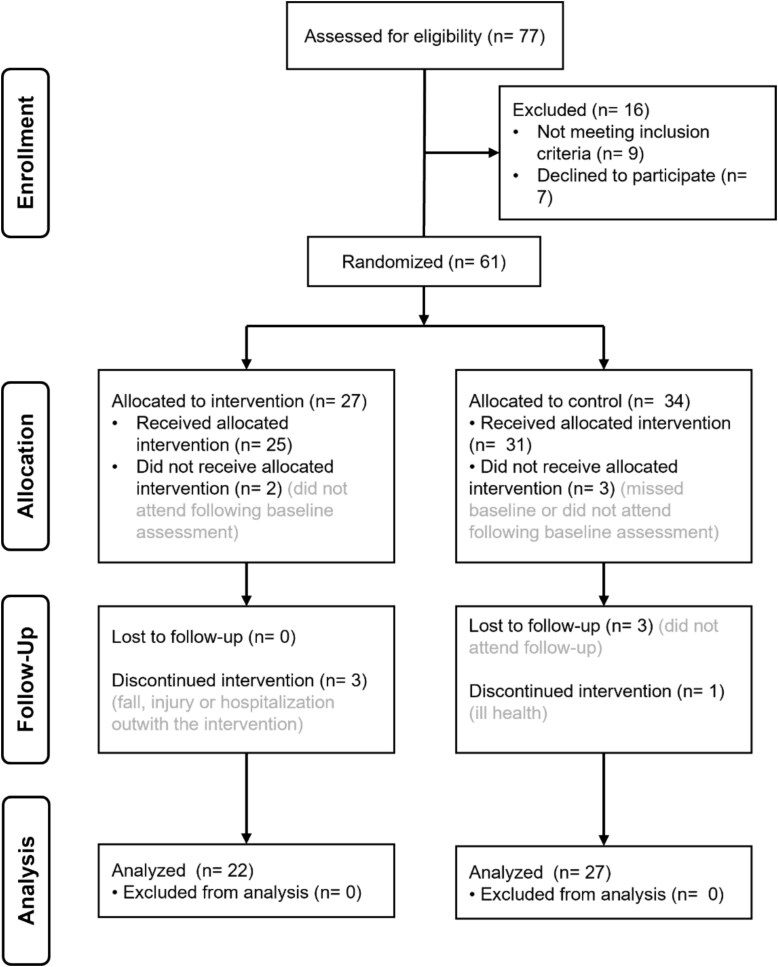
Consolidated Standards of Reporting Trials (CONSORT) flow diagram showing participant recruitment, allocation, and attrition throughout the study.

Sixty-one participants (79% of those interested) were recruited (Figure 2), five of whom did not receive the intervention. Four participants withdrew (three intervention, one control) due to unrelated health issues or hospitalisation, and three were lost to follow-up. Data analysis was conducted on 49 participants (22 FRT, 27 control).

Although originally planned for 6 weeks, the intervention was reduced to 5 weeks due to a rapid improvement and plateau in floor-rise ability. This decision was made to avoid unnecessary burden on participants and did not affect the planned outcome assessments. Session attendance was high (overall: 86%; FRT: 89%; control: 86%). Some participants completed the intervention in three or four sessions due to early proficiency (see Supplementary Appendix 3 for the full adherence data). The BCM was successfully modified for three participants with restricted knee flexion secondary to double knee replacements, to bypass the ‘half-kneeling’ phase (see Supplementary Appendix 4 for more details on the modified protocol).

### Baseline characteristics and safety

The control group was significantly older and had attended Otago classes for a shorter duration than the FRT group (Table 1), though their average attendance was still 2 years. There were no significant baseline differences between groups for any outcome measures (Table 2). No adverse events occurred during the intervention, and the lifting cushion was not required.

**Table 1:** Baseline characteristics of participants in the FRT and control groups

	FRT	Control	*P* values
Sample size	22	27	
Age (years)^†^	74.7 (5.1)	78.4 (5.7)	0.024^*^
Number of male participants	5 (23%)	2 (7.4%)	
Number of falls in previous year ^†^^†^	0 (0–3)	0 (0–3)	**0.695**
Number of diagnosed medical conditions^†^^†^	2 (0–4)	2 (0–6)	**0.513**
Number using walking aids indoors	0 (0%)	1 (4%)	
Number using walking aids outdoors	3 (14%)	5 (19%)	
Number of years attending Otago class^†^^†^	3 (0–10)	2 (0–6)	0.011^*^

Data presented as frequency (percentage) except ^†^ mean (sd) with T-test and ^†^^†^ median (range) and % with Mann–Whitney test where indicated.

**Table 2:** A summary table of baseline and post-intervention values for various outcome measures. Lower scores indicate improvement except with PAMF, where higher scores reflect greater perceived ability to manage risk of falls. Timed floor-rises (from half-kneeling, side-sitting and supine) are reported in seconds

		Baseline			Post-intervention			Interaction analysis (Group × Time)		
Test	Group	*n*	Mean (95% CI)	Between-group *P*	*n*	Mean (95% CI)	Between-group *P*	Mean difference (95% CI)	Effect size (ƞ_p_^2^)	Interaction *P*
**Questionnaires and VAS Scales**										
FES-I	FRT	22	25.5 (21.6, 29.5)	0.171	22	23.8 (20.4, 27.2)	0.384	−2.0 (−6.6, 2.6)	0.016	0.527
	Control	27	26.4 (22.8, 30.0)		27	25.8 (22.7, 28.9)				
FoF VAS	FRT	22	31.5 (20.8, 42.3)	0.218	22	26.5 (15.4, 37.5)	0.320	−7.5 (−22.4, 7.5)	0.021	0.624
(indoor)	Control	27	35.7 (26.6, 45.3)		27	33.9 (23.9, 43.9)				
FoF VAS	FRT	22	47.4 (35.3, 59.6)	0.126	22	33.5 (20.9, 46.2)	0.076	−15.4 (−32.4, 1.9)	0.065	0.098
(outdoor)	Control	27	50.1 (39.2, 61.1)		27	48.9 (37.5, 60.4)				
Avoidance VAS	FRT	22	29.1 (18.1, 40.2)	0.285	22	23.5 (11.5, 35.3)	0.339	−7.7 (−23.7,8.3)	0.019	0.631
	Control	27	40.3 (30.3, 50.3)		27	31.1 (20.4, 41.9)				
PAMF	FRT	22	13.6 (12.4, 14.8)	0.498	22	16.3 (14.9, 17.7)	**0.011**	2.5 (0.6, 4.3)	0.129	**0.033**
	Control	27	12.9 (11.8, 14.0)		27	13.8 (12.6, 15.1)				
**Timed floor-rise**										
Half-kneeling	FRT	20	3.9 (2.7, 5.2)	0.372	22	1.5 (1.0, 2.0)	**<0.001** ^*^	−2.0 (−2.7, −1.3)	0.470	**<0.001** ^*^
(seconds)	Control	24	4.5 (3.3, 5.7)		23	3.5 (3.0, 4.0)				
Side-sitting	FRT	19	8.0 (6.0, 9.9)	0.228	22	4.6 (3.0, 6.2)	**<0.001** ^*^	−4.0 (−6.3, −1.7)	0.254	**0.046** ^*^
(seconds)	Control	21	9.8 (7.9, 11.7)		22	8.6 (7.0, 10.2)				
Supine	FRT	17	13.1 (10.6, 15.5)	0.352	22	7.1 (5.4, 8.7)	**0.002** ^*^ ^,^ ^†^	−4.2 (−6.7, −1.8)	0.296	**0.001** ^*^ ^,^ ^†^
(seconds)	Control	17	11.6 (8.9, 14.3)		17	11.3 (9.5, 13.1)				

Abbreviations: FRT: floor-rise training; FES-I: falls efficacy scale—international, FoF: fear of falling; VAS: visual analogue scale; PAMF: perceived ability to manage risk of falls or actual falls; ƞ_p_^2^: partial eta squared.

^*^Interaction based on log transformed data.

^†^log transformation did not correct lack of equality of error variances. Sig = p value. n = sample size.

### Fear of falling and activity avoidance

There were no significant interactions between time point and group for the primary outcome (FES-I), FoF VAS (indoor/outdoor) or activity avoidance VAS, with no between-group differences post-intervention (Table 2).

### Perceived ability to manage risk of falls or actual falls

In the primary analysis, PAMF scores showed a significant interaction between time point and group (*P* = .033). Post-intervention, the FRT group reported significantly higher perceived ability than controls (mean difference 2.5; 95% CI: 0.6, 4.3; medium effect *ƞ_p_*^2^ = 0.129, *P* = .011).

### Floor-rise ability

At baseline, 77% of the FRT group could rise independently from supine, increasing to 100% post-intervention. In contrast, independent supine floor-rise in the control group remained unchanged at 63% (see Supplementary Appendix 5 for more data on floor-rise ability). After adjusting for age, Otago duration and baseline ability, the intervention significantly increased the probability of rising from the supine position by 27% compared with controls (adjusted RD = 27%, 95% CI 7% to 47%, *P* = .007). Positive trends were also observed for rising from side-sitting (RD = 11%, *P* = .14) and half-kneeling (RD = 9%, *P* = .16), though these did not reach statistical significance, likely due to ceiling effects.

### Floor-rise timing

A significant interaction was found across all three positions (Table 2). The FRT group demonstrated significantly faster rise times post-intervention compared to controls. Effect sizes for these differences were all large, ranging from *ƞ_p_*^2^ = 0.254 (side-sitting) to *ƞ_p_*^2^ = 0.470 (half-kneeling). Log transformation did not fully correct for inequality of variance in supine rise times (Levene’s test, *P* < .05), requiring cautious interpretation of these results.

### Sensitivity analysis

A secondary sensitivity analysis (ANCOVA) was performed to adjust for the baseline imbalances in age and Otago duration. This confirmed that no significant interaction existed for FES-I or VAS measures. Additionally, the significant interaction for both half-kneeling and supine timed floor-rise remained. However, the interaction for side-sitting timing became marginally non-significant (*P* = .053). Notably, the interaction for PAMF became non-significant (*P* = .195), apparently due to a significant association between age and this measure for the post-measurement time point (*P* = .002, *ƞ_p_*^2^ = 0.195).

### Focus group findings

A total of 15 participants attended the post-intervention focus groups (FRT *n* = 7; Control *n* = 8). The findings are structured according to the three key areas of inquiry.

### Intervention acceptability

Participants in the FRT group reported that the intervention was enjoyable, improved with practice and increased their confidence.

‘I think it was a case of practice made perfect. The more you did it, then you got more into the way of it’.

‘I definitely improved from start to finish. At the beginning I wasn’t very good. By the end I could get down and back up again. It definitely helped me’.

Initial concern regarding the descent and ascent was a significant barrier. The transition from half-kneeling to standing was identified as the most physically demanding aspect, with some reporting knee soreness.

‘I thought if I got down, I’m not going to get back up again because I cannot bend my knees. They just won’t bend. So that was a big concern’.

However, facilitators included the presence of the instructor and the lifting cushion.

‘I thought I need to try... I knew that help was there and encouragement.’

Among the control group, the NHS Inform videos were well-received and considered informative, with some participants practicing FRT at home. Suggestions included adding content on managing the ‘shock factor’ after a fall.

### Perceived impact

Participants in both groups reported limited prior FoF, though some indicated increased confidence post-intervention.

‘I wasn’t really concerned about falling, but I’ve no concerns now because I think, right, I can get up’.

Qualitative data revealed a distinction in fear: for many, the fear was not of the fall event, but of physical damage.

‘I think the only concern I would have about falling is back to the injury, it’s not the falling per se, it’s if you end up breaking something’.

### Trial process and feasibility

Participants found the recruitment process fair and non-coercive. Feedback regarding outcome measures revealed a specific preference for the multi-item scales (FES-I and PAMF) over the single-item VAS. Participants reported that the specific questions in the longer scales were easier to interpret and answer than the broad nature of the VAS items.

## Discussion

This study found that five FRT sessions did not significantly impact the primary outcome (FoF) or activity avoidance compared to the controls. However, FRT significantly improved floor-rise performance (faster rise times and improved independent ability) and perceived post-fall efficacy (increased PAMF scores).

The lack of significant change in FES-I scores likely reflects two converging factors. First, there is a conceptual distinction between the intervention’s target and the outcome measure. FRT targets post-fall management, whereas the FES-I primarily measures pre-fall efficacy (confidence in preventing the fall event) [39]. As FRT does not improve balance or prevent the fall itself, its impact on pre-fall confidence is indirect and likely smaller than primary prevention strategies.

Second, any indirect benefit to FES-I scores, mediated by reducing the specific fear of the ‘long-lie,’ was likely masked by a floor effect. Participants entered the study with ‘Moderate’ baseline concern (mean FES-I = 25.5–26.4) [48], largely attributable to their long-term engagement (mean 2–3 years) in Otago classes, a programme known to reduce FES-I [26]. Qualitative data supports this interpretation; participants noted, ‘I wasn’t really concerned about falling, but I’ve no concerns now’ suggesting that while residual anxiety was eliminated, baseline levels were insufficiently high to reach statistical significance. This reinforces the value of the PAMF, which successfully captured the specific gain in post-fall efficacy.

Previous research improving floor-rise ability used a wide range of training dosages (6–36 sessions) [17, 21–23]. At the lower end of this range, Hofmeyer *et al.* [29] demonstrated efficacy with six 45-minute sessions (270 minutes total). However, their protocol required resource-intensive one-to-one training under physiotherapist supervision and a complex, individualised strategy rather than a standardised method.

In contrast, the current study is the first to demonstrate that a significantly lower volume (three to five 20-minute sessions; 60–100 minutes total) of FRT alone can provide substantial functional improvements in the floor-rise skill. Unlike previous individualised models, this study utilised the standardised BCM. This suggests that FRT is scalable and can be effectively delivered by Postural Stability Instructors without individual physiotherapy assessment, addressing a critical workforce barrier. Notably, all intervention participants who could not independently rise at baseline were able to do so post-intervention, reinforcing the functional value of this brief, scalable intervention.

An unexpected trend toward improvement in the control group may be attributed to self-directed practice. Several control participants reported attempting FRT at home after watching the videos. They also praised the videos for being informative and suggested they be promoted more widely by healthcare providers. While this represents a limitation in study control, it highlights that video-based instruction offers value. However, this did not translate to increased functional success (binary ability to rise), suggesting that while videos improve awareness, supervised practice is necessary for skill acquisition.

Given that a significant proportion of older adults cannot floor-rise independently following a fall [9], the brief FRT intervention demonstrated here offers a strategy to promote independence and prevent harm. Since many older adults who call an ambulance after a fall do not require hospitalisation [12, 49], equipping them with the skill to rise could significantly reduce healthcare resource use. Crucially, these findings support adding FRT to existing fall prevention programmes, as the participants were already engaged in such services.

Future research should explore the effectiveness of FRT in older adults not already engaged in falls prevention classes. A planned definitive study will explore if a similar improvement in floor-rise ability can be achieved within those most at risk of a long-lie. Given that PAMF and FES-I had a high acceptability in our cohort, these outcome measures remain appropriate for future trials.

### Limitations

Several limitations exist. Assessments were conducted by the intervention provider, though objective measures were used where possible. Sensitivity analysis indicated that the age imbalance between groups affected specific secondary results (e.g. PAMF interaction), suggesting post-fall efficacy is age-dependent; future studies require rigorous age stratification. Moderate baseline FoF may have created a floor effect, reducing the likelihood of detecting significant between-group differences. Finally, as participants were recruited from weekly falls prevention classes, findings may lack external validity for the general older adult population.

## Conclusion

Although a brief FRT intervention (3–5 sessions) did not significantly reduce FoF in older adults already attending community-based falls prevention classes, it significantly improved floor-rise time, ability and PAMF. Specifically, the intervention successfully enabled 100% of the intervention group to rise independently. Given the high burden of long-lies and ambulance callouts, these findings support the integration of FRT into existing falls prevention programmes. If future research determines FRT to be effective and implementable in those most at risk of long-lies, it may prove a critical step toward reducing fall-related harm and healthcare costs.

## Supplementary Material

aa-25-2196-File002_afag064

## Research Data Transparency and Availability:

The anonymised data supporting the findings of this study are available from the corresponding author upon reasonable request.
