# Supplementary material for: Comparing the impact of active floor-rise training with video demonstration on fear of falling and independent floor-rise ability in older adults living in the community: a pilot cluster randomised controlled trial
Source: Age Ageing. 2026 Mar 27;55(3):afag064. doi: 10.1093/ageing/afag064 (PMC13023370; doi:10.1093/ageing/afag064)
Supplement: aa-25-2196-File002_afag064 [file aa-25-2196-file002_afag064.pdf]

Supplementary Data

Comparing the Impact of Active Floor-Rise Training with Video Demonstration on Fear of Falling and Independent Floor-Rise Ability in Older Adults Living in the Community: A Pilot Cluster Randomised Controlled Trial.

Table of Contents

Appendix 1: Floor-Rise Training Instructions 1

Appendix 2: Semi-Structured Interview Prompts 1

Appendix 3: Number of Completed Sessions and Attendance 2

Appendix 4: Description of Modified Floor-Rise Technique 2

Appendix 5: Number of participants that can Rise from Different Positions 3

References 3

Appendix 1: Floor-Rise Training Instructions

Table a) FRT instructions adapted from Reece and Simpson [1].

	BCM Instructions
1	Turn to face your chair from a few steps away. Step forward with your strongest leg. Place your hands on the arms (or the seat) of the chair. Lean your weight evenly over the chair so that it does not tip. Bring both feet together and stand in front of the chair.
2	As per step 1, then slowly lower your back knee to the floor. Make sure you also bend your front knee at the same time. Untuck the toes of the rear foot. To finish the movement, re-tuck the toes of the rear foot and stand using the chair.
3	As per step 2, then lower the remaining knee to the floor into a high kneeling position. Once achieved, bring your strongest leg forward. Once achieved, cycle back through the previous steps.
4	As per step 3 and then lower both hands to the floor, one at a time, to come to a four-point kneeling position. Walk away from the chair in four-point kneeling to prevent head contact with the chair in subsequent steps. Once achieved, cycle back through the previous steps.
5	As per step 4, then lower hips/bottom gently down onto the floor. Once this position is achieved, lean weight over hands and lift bottom up and over knees in a four-points kneeling. Once achieved, cycle back through the previous steps.
6	As per step 5, then lower the body to a side-lying position first onto an elbow and then onto a shoulder. Using the top arm for assistance, push up back into sitting onto an elbow and then onto hands. Once achieved, cycle back through the previous steps.
7	As per step 6, turn from side-lying to lying on your back. Using the legs for assistance, twist back into side-lying. Once achieved, cycle back through the previous steps.

Appendix 2: Semi-Structured Interview Prompts

Table b) Outline for the semi-structured interview

	Semi-Structured Interview Prompts
1	Views about falls
2	Views about FRT or NHS inform videos
3	Views about each step of FRT (FRT group only)
4	Views about preferred ways of getting up off of the floor outwith FRT (FRT group only)
5	Views about how the intervention/videos changed concern of falling.
6	Views about what is missing from NHS inform videos (control group only)
7	Views about how NHS inform videos can be improved (control group only)
8	Views about assessment and outcome measures
9	Views about recruitment, enrolment and participation

Appendix 3: Number of Completed Sessions and Attendance

Table c) The number of completed FRT or control sessions and overall attendance.

	Total	FRT	Control
Number of sessions offered	5 (3-5)	5 (3-5)	5 (0)
Number of sessions attended	4 (1-5)	4 (3-5)	5 (1-5)
Attendance of offered sessions (%)	86%	89%	84%

Data presented as median (range) and % where indicated.

Appendix 4: Description of Modified Floor-Rise Technique

Three participants had highly restricted knee flexion secondary to double knee replacements. The backwards-chaining method technique was modified for these individuals to bypass the "half-kneeling" phase. This involved placing their forearms on the chair, walking the feet back and lowering the knees down together in a more extended position (see Figure a). This was done in reverse to rise from the floor and worked reliably in participants with double knee replacements.

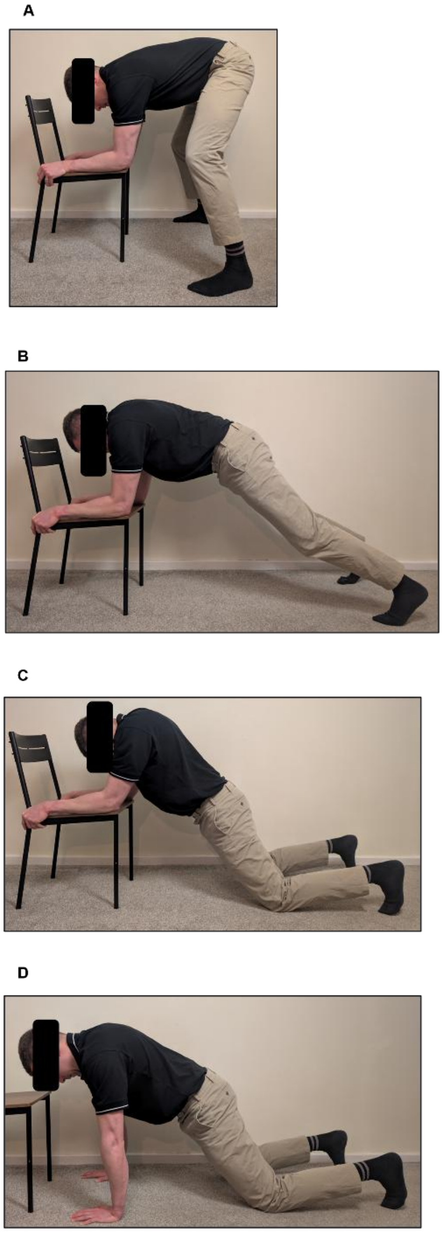

Figure a) Images of the four positions used during floor-rise training for participants with limited knee flexion to bypass the half-kneeling phase.

Appendix 5: Number of Participants that can Rise from Different Positions

Table d) Number and percentage of participants that can rise from different positions.

Rise Position		FRT	Control
Half-Kneeling	Baseline	20 (91%)	24 (89%)
	Post-Intervention	22 (100%)	23 (85%)
Side-Sitting	Baseline	19 (86%)	21 (78%)
	Post-Intervention	22 (100%)	22 (81%)
Supine	Baseline	17 (77%)	17 (63%)
	Post-Intervention	22 (100%)	17 (63%)

References

1. Reece AC, Simpson JM. Preparing older people to cope after a fall. Physiotherapy. 1996 Apr 1;82(4):227-35.
